# Gender differences in the association between adherence to healthy diet principles and adherence to cardiopreventive medication among adults from Québec (Canada)

**DOI:** 10.1017/S0007114525000030

**Published:** 2025-02-14

**Authors:** Lise Leblay, Jacob Lessard-Lord, Jean-Sébastien Paquette, Line Guénette, Jean-Philippe Drouin-Chartier

**Affiliations:** 1 Centre Nutrition, Santé et Société (NUTRISS), Institut sur la Nutrition et les Aliments Fonctionnels (INAF), Université Laval, Québec, Canada; 2 Faculté de Pharmacie, Université Laval, Québec, Canada; 3 Département de médecine familiale et de médecine d’urgence, Faculté de Médecine, Université Laval, Québec, Canada; 4 VITAM, Centre de recherche en santé durable, Université Laval, Québec, Canada; 5 Groupe de médecine de famille universitaire du Nord de Lanaudière, CISSS Lanaudière, Saint-Charles-Borromée, Québec, Canada; 6 Centre de recherche du CHU de Québec, Axe Santé des populations et pratiques optimales en santé, Université Laval, Québec, Canada

**Keywords:** Gender, Adherence behaviour, Healthy diet principles, Cardiopreventive medication

## Abstract

Adherence to healthy diet principles and to cardiopreventive medication, both key behaviours in CVD prevention, is known to differ between women and men. Whether these adherence behaviours are differentially related among women and men has never been thoroughly assessed. The objective was to assess gender differences in the association between adherence to healthy diet principles and to cardiopreventive medication in adults free of CVD. This cross-sectional study included 268 women and 204 men from the CARTaGENE cohort (Québec, Canada) who were using antihypertensive and/or cholesterol-lowering medication. Adherence to healthy diet principles was assessed using the Alternate Healthy Eating Index (AHEI, %), calculated from a validated FFQ assessing diet in the 12-month preceding its completion. Medication adherence was assessed using the daily pharmacotherapy possession rate (DPPR, %), calculated from prescription claim data over the same 12-month period. In multivariable-adjusted analyses, an inverse association between AHEI and DPPR was observed among men (*β*_AHEI_ for 10 % increment in DPPR = –0·65 %; 95 % CI −1·28 %, −0·03 %; *P* = 0·04), while it tended to be positive among women (*β* = 0·44 %; 95 % CI −0·11 %, 1·00 %; *P* = 0·12; *P*_gender×DPPR_ = 0·01). The negative association between AHEI and DPPR was stronger among men who never smoked or used cholesterol-lowering medication only. Among women, the positive association was stronger and statistically significant among those with obesity or using ≥ 3 medications simultaneously. Association between adherence to healthy diet principles and to cardiopreventive medication differs between women and men, with men potentially facing greater challenges in achieving optimal complementarity between these two behaviours.

CVD are the leading causes of death worldwide. According to the WHO, 17·9 million individuals died from CVD in 2019, which represented 32 % of all deaths on a global scale^(1)^. Hypertension and dyslipidemia (e.g. high blood cholesterol) are both major modifiable risk factors for CVD^(2,3)^. Management of these risk factors relies on the complementary use of lifestyle modification, for which diet is a key component, and medication^(4,5)^. Multiple randomised controlled trials demonstrated that adhering to healthy diet principles – such as consuming diets low in red and processed meats and ultra-processed foods, and high in minimally processed plant foods – can lower blood pressure (BP) or LDL-cholesterol to an extent similar to that of first-line BP- or cholesterol-lowering drugs^(6–8)^. Also, improving diet quality has been demonstrated as an effective strategy to reduce not only medication intensity but also the need for medication^(9)^. Still, real-world data showing a lack of complementarity between dietary and pharmacological management of CVD risk are accumulating^(10)^. For instance, data from the National Health and Nutrition Examination Survey showed that, over the first decade of the 2000s in the USA, energy and fat intakes steadily increased among individuals using a statin, while remaining stable among non-users^(11)^. Similarly, our group recently highlighted the lack of complementarity between diet quality and the intensity of cholesterol-, BP- and glucose-lowering medications in adults from Québec (Canada) ^(12–14)^. We observed that, among adults with metabolic syndrome, statin use was associated with lower diet quality^(12)^. Additionally, in young adults with hypertension or type 2 diabetes, the intensity of both BP- and glucose-lowering medications was inversely associated with diet quality^(13,14)^.

In the aforementioned studies, diet quality indices (e.g. Alternate Healthy Eating Index (AHEI)) served as metrics of overall adherence to healthy diet principles, but adherence to medication was not assessed. To our knowledge, the literature on the association between these two adherence behaviours is limited to only two cross-sectional studies. One study was conducted in Australia among 270 adults with treated hypertension and the other from South Korea among 417 adults in secondary prevention of myocardial infarction^(15,16)^. Both reported that adherence to healthy diet principles was positively associated with adherence to medication. Still, none of the studies assessed whether the association between adherence to healthy diet principles and adherence to medication differed due to gender. On the one hand, greater adherence to healthy diet principles among women relative to men has been repeatedly reported in many countries^(17–19)^. On the other hand, adherence to cardiopreventive medication is known to be lower among women^(20–23)^. This could be due to a greater preference towards lifestyle modification relative to medication interventions among women^(24)^. However, how these differences translate into how women and men with prevalent CVD risk factors concomitantly adhere to both healthy diet principles and medication remains unknown. These data are necessary to develop gender-specific interventions that promote long-term adherence to healthy diet principles and medication for optimal primary prevention of CVD.

The objective of this study was to assess gender differences in the association between adherence to healthy diet principles and adherence to cardiopreventive medication (i.e. cholesterol- and/or BP-lowering drugs) within a cohort of adults with hypertension and/or dyslipidemia from Québec (Canada). As the primary outcome of this study, we assessed the differential association between the AHEI, reflecting adherence to cardiopreventive dietary habits, and the pharmacotherapy possession rate, an indicator of adherence to cardiopreventive medications, both evaluated over an overlapping 12-month period, according to gender. Second, we investigated whether the association between these two adherence behaviours differed according to specific sociodemographic and clinical characteristics (e.g. education level, smoking status, type and number of medications) among women and men, separately. The focus on BP- and cholesterol-lowering medications in the current study was motivated by the fact that the use of these drugs is recommended, in complement to similar dietary modification, to manage BP or dyslipidemia in primary prevention of CVD.

## Methods

The study protocol was reviewed and approved by the Laval University Ethics Committee, the CARTaGENE Sample and Data Access Committee and the Québec Statistics Institute.

### Study population

This study is a cross-sectional analysis conducted within the CARTaGENE Québec population-based cohort (Phase A)^(25)^. In 2009–2010, residents from the province of Québec (Canada), aged 40–69 years, and residing in metropolitan areas representing 56 % of the Québec population, were randomly solicited from provincial health insurance registries to participate in CARTaGENE. Recruitment was stratified according to 2006 census data based on age, sex and area of residence. All participants (*n* 19 069) signed an informed consent form. CARTaGENE adhered to the principles of the Declaration of Helsinki.

Baseline data collection was completed during an in-person visit (2009–2010). The visit comprised the completion of a self-administered sociodemographic and lifestyle questionnaire^(26)^, an interviewer-administered health questionnaire, physical measurements and biospecimen collection. In 2012, participants were invited to complete a FFQ from home, which was completed and mailed back by about 10 000 individuals. The FFQ was the Canadian Dietary History Questionnaire II (CDHQII), which assessed diet over the 12 months preceding its completion (fully described below)^(27,28)^.

For the present study, information on medication was obtained from the Québec Public Prescription Drug Insurance Plan database. Adults who are eligible for this insurance plan are those (1) aged less than 65 years and who do not have access to a private prescription drug insurance plan (e.g. group insurance plan and employee benefits plan), (2) aged ≥ 65 years or (3) recipients of a financial assistance programme^(29)^. Most individuals covered by the Québec Public Prescription Drug Insurance Plan fall under the category of being 65 years of age and over. The coverage is the same, regardless of the eligibility category of the individual. The Québec Public Prescription Drug Insurance Plan database contains information on the type of coverage, period of coverage, as well as on all prescription drug claims (i.e. drug name, claim date, quantity supplied and duration of supply), for each covered individual. This database is recognised for its accuracy and comprehensiveness in tracking prescription drugs^(30,31)^. To obtain information on diagnostics/procedures (per International Classification of Diseases, 9th and 10th revisions) and hospitalisations that occurred before FFQ completion, we sourced the Québec Maintenance and Exploitation of Data for the Study of Hospital Clientele (MED-ECHO) database^(32)^. This database contains these data for all individuals enrolled in the Québec universal healthcare programme, which encompasses approximately 99 % of the Québec population^(33)^. Both databases were linked with the individual participants’ data within the CARTaGENE dataset.

In the current analysis, adherence to cardiopreventive medication was evaluated over the same time frame as was assessed diet, that is, the 12-month period preceding FFQ completion (fully explained below). Therefore, among participants who returned the FFQ, we included those who: (1) adequately completed the FFQ (i.e. < 40 % of blank items); (2) reported plausible energy intakes in the FFQ (i.e. women: 500–3500 kcal/d; men: 800–4200 kcal/d); (3) were covered by the Québec Public Prescription Drug Insurance Plan for the entirety of the 365-d period preceding FFQ completion date; and (4) had filled a prescription for at least one cardiopreventive medication (i.e. BP- or cholesterol-lowering medication) that duration of supply overlapped with the 365th day before the date of completion of the FFQ. The latter ensured that pharmacological treatment for arterial hypertension or dyslipidemia was initiated before, and not during, the 12-month period preceding FFQ completion, so that adherence to cardiopreventive medication was evaluated over the same time frame as was assessed diet. Finally, we excluded participants with a personal history of diabetes, CVD or cancer prior to FFQ completion to focus the study on the early stages of disease prevention through both diet and medication. A total of 472 individuals (268 women and 204 men) were included in the study (online Supplementary Figure S1).

### Assessment of diet and adherence to healthy diet principles

Diet was assessed using the CDHQII^(27,28–34)^. The CDHQII is a FFQ that assesses the frequency of consumption of 153 foods and the portion size usually consumed in the 12 months preceding its completion. It was initially developed and validated by the US National Cancer Institute^(28,36–38)^. The US version has been modified to reflect food availability, brand names, nutrition composition and food fortification in Canada.

Adherence to healthy diet principles was assessed using the 2010 AHEI^(39)^. The AHEI is calculated based on intakes of foods and nutrients that have been consistently associated with lower risk of CVD. Intakes of vegetables, fruits, whole grains, nuts and legumes, fatty fish and PUFA are positively scored. Intakes of red and processed meat, sugar-sweetened beverages and fruit juices, *trans*-fat, and Na are negatively scored. A score from 0 (worst) to 10 (best) was assigned to each component based on *a priori* determined thresholds, except for Na, for which scoring is based on deciles of intake. The score of each component was summed to obtain the total AHEI ranging from 0 (minimal adherence) to 100 points (maximum adherence). In the current study, the AHEI was calculated without the 11th component on alcohol. Instead, alcohol consumption was treated as a separate behaviour, distinct from adherence to healthy dietary principles, and was included as a covariable in the analyses (see *Statistical analyses*).

### Assessment of medication and adherence to cardiopreventive medication

Information on cardiopreventive medication (i.e. BP- and/or cholesterol-lowering agents) was extracted from the Québec Public Prescription Drug Insurance Plan database (RAMQ). For all included participants, the use of antihypertensives and/or cholesterol-lowering agents was first identified using the American Hospital Formulary Service (AHFS) classification. Subsequently, the Canadian Drug Identification Numbers (DIN) were used to identify each medication, that is, the specific combination of one or more active pharmaceutical ingredients and the corresponding dosages within a single pill^(40)^.

To calculate medication adherence, data on each medication claimed by participants over the 365-d period preceding FFQ completion were extracted. These data included: (1) information on the medication (i.e. AHFS class, DIN and dosage), (2) dispensation dates (i.e. all dates the participant claimed the medication at a pharmacy) and (3) duration of supply (i.e. the estimated number of days covered by the dispensation according to direction for use at each claim). Next, for each medication, based on dispensation dates and duration of supply, we determined whether the medication was available to the participants daily, using standard procedures^(41)^. For instance, if two consecutive claims for the same medication, both with 30 d of supply, were separated by 45 d, the medication was available to the participant for only 45 d over the 60 d following the first claim (online Supplementary Figure S2A, case 1). In cases where the medication was resupplied before the end of the previous supply, the dispensation date was modified for the first day following the end of the previous claim. For instance, if two consecutive claims for the same medication, both with 30 d of supply, were separated by 25 d, the medication was considered available to the participant for each of the 60 d following the first claim (online Supplementary Figure S2A, case 2). Finally, medication claims were used to identify individuals whose cardiopreventive pharmacotherapy remained stable or changed over the 12-month study period. Changes in pharmacotherapy could include initiation of an additional medication, adjustment in dosage or replacement of a medication with another. In cases where, during the last supplied period of a medication, another drug with the same indication was claimed for the first time, the former medication was considered to be replaced by the latter. For instance, if atorvastatin was available until day 30, and rosuvastatin was first claimed on day 30, then atorvastatin was considered as replaced by rosuvastatin (online Supplementary Figure S2A, case 3). However, in cases where a medication was no longer resupplied over the study period, without evidence of substitution, these cases were treated as non-adherence because both BP- and cholesterol-lowering drugs are meant to be used chronically (online Supplementary Figure S2A, case 4). Finally, in the present study, the number of cardiopreventive medications available at baseline referred to the count on the 365th day before FFQ completion. The maximum number of medications was determined from the highest count available on a single day over the 12-month study period. Pharmacy claim data management was performed using RStudio (version 2023.09.1.0).

Adherence to cardiopreventive medication was assessed as the average daily pharmacotherapy possession rate (DPPR, %), calculated over the 12 months preceding FFQ completion^(41)^. The DPPR method involves calculating daily possession rates for all claimed medications and weighting them by the number of medications expected to be available each day. For instance, if, on a specific day, an individual had only two medications available out of three medications claimed due to late resupply for the third one, the calculated daily possession rate was 66 % (i.e. 2/3) (online Supplementary Figure S2B). These daily rates were then averaged to obtain the yearly average DPPR over the 365-d period preceding FFQ completion. Higher yearly DPPR is a validated proxy for higher adherence to medication^(41,42)^. In cases where included participants were hospitalised during the 12 months preceding FFQ completion, the hospitalisation period (identified through admission and discharge dates from the MED-ECHO database) was deducted from the yearly DPPR calculation, meaning that the DPPR was calculated over a period of < 365 d, as done in previous studies^(42–44)^.

In sensitivity analyses, we used two alternative methods to calculate medication adherence. The first approach, referred to as *full pharmacotherapy availability* in subsequent sections, was more stringent, based on the rationale that individuals need to adhere to their full pharmacotherapy for it to be effective. Thus, daily possession rates < 100 % were recoded as 0 %^(42)^. Conversely, the other approach, referred to as *partial pharmacotherapy availability* in subsequent sections, was less stringent and considered that having at least one available medication was sufficient to be considered adherent to the whole pharmacotherapy. Thus, daily possession rates > 0 % were recoded as 100 %^(42)^. These alternative approaches are commonly used in pharmacoepidemiology^(42)^. Overall, the primary and sensitivity approaches to calculating medication adherence provide complementary insights, accounting for different perspectives on how adherence to medication is defined. The calculation of adherence was performed using SAS Studio software (version 3.5).

### Assessment of covariables

Participants’ sex was reported in a binary fashion (female and male) in CARTaGENE health questionnaire during the baseline in-person interview. Participants’ gender was not distinctly queried. In the present study, we used information on sex as a proxy of gender (women and men). Data on age, household annual income, smoking status and level of physical activity were collected through the self-administered questionnaire during the in-person interview. Physical activity was assessed using the International Physical Activity Questionnaire^(45)^. Anthropometry was measured by a research associate during the interviews. Waist circumference was measured twice (SECA 200 measuring tape), as was the participants’ height (SECA 214 portable stadiometer). A digital scale was used to measure the participant’s weight. Data on alcohol consumption (g/d) and energy intake (kcal/d) were calculated from CDHQII data.

### Statistical analyses

Statistical analyses were conducted using SAS Studio software (version 3.5). An *a priori* power calculation could not be conducted because no prior study has assessed the association between the AHEI and the DPPR, leaving no data to inform potential strength of the association. All statistical tests were two-sided with a significance threshold set at *P* < 0·05. First, we compared adherence to both healthy diet principles (i.e. AHEI, in percent) and cardiopreventive medication (i.e. yearly DPPR, in percent) according to participants’ gender, using linear regression models (GLM procedure). AHEI and DPPR were included as dependent variables in distinct models. Models were adjusted for age (years), annual household income (<$50 000; $50 000 < $100 000; ≥ $100 000), BMI (kg/m^2^), smoking status (never, past and current), physical activity level (low, moderate and high), alcohol consumption (grams/d), energy intake (kcal/d) and the type of cardiopreventive medication used during study (antihypertensive medication only *v*. cholesterol-lowering medication only *v*. both). Mutual adjustment for DPPR (%) or AHEI (%) was used depending on the dependent variable.

The primary analyses aimed to determine whether the association between adherence to healthy diet principles and adherence to cardiopreventive medication differed between women and men. To evaluate this, we used general multiple linear models with AHEI (total score and sub-scores, sequentially) as the dependent variable, and the interaction term between DPPR and gender as the main independent variable. These models were adjusted for age, annual household income, BMI, smoking status, physical activity level, alcohol consumption, energy intake and pharmacotherapy type (all fixed effect covariables). In analyses with AHEI sub-scores as dependent variables, we mutually adjusted for each sub-score. Evidence of a differential association due to gender was assessed using the *P*-value of the interaction term between DPPR and gender. In sensitivity analyses, we repeated these analyses using the two alternative calculation methods for DPPR, that is, full pharmacotherapy availability and partial pharmacotherapy availability, sequentially. We also compared, in a gender-specific fashion, adherence to healthy diet principles according to three recognised thresholds for adherence to cardiopreventive medication: DPPR < 20 %, reflecting non-adherence; DPPR between 20 % and < 80 %, reflecting partial adherence; and DPPR ≥ 80 %, reflecting adequate adherence^(46)^.

Next, we conducted pre-specified stratified analyses to assess whether the gender-specific association between AHEI and DPPR differed according to education level (high school or less *v*. college or university), annual household income (< $50 000 *v*. ≥ $50 000), BMI (< 30 kg/m^2^ *v*. ≥ 30 kg/m^2^), smoking status (never *v*. past *v*. current), pharmacotherapy type (antihypertensive medication only *v*. cholesterol-lowering medication only *v*. both) and maximum number of cardiopreventive medications dispensed during the study (≤ 2 medications *v*. > 3 medications and stability of cardiopreventive medication over the preceding 12 months (stable *v*. not stable). Again, the *P*-value of the interaction term between DPPR and the stratification variable was used to inform on gender-specific subgroup differences.

For all statistical models, the normality of the linear models was assessed using the distribution of the scaled residual values. In cases where these values were not normally distributed, we used the Box-Cox approach (TRANSREG procedure) to identify the transformation that allowed the normalisation of the model-scaled residual values. If the transformation failed to normalise the model, a rank transformation (RANK procedure) was used. To facilitate interpretation of models that required transformation, tables show untransformed *β* coefficients and CI with *P*-values calculated from the transformed model.

## Results

Characteristics of the 268 women and 204 men included in the study are presented in Table 1. Women and men had similar ages and BMI. Annual household income distribution was also similar, with both women and men more likely to have an annual household income of < $50 000. While women were more likely to have never smoked, men were more likely to be past smokers. Physical activity level distribution was also similar between women and men, with most individuals with moderate or high levels. Alcohol and energy intake were higher among men. As expected, most participants in both groups were covered by the Québec Public Prescription Drug Insurance Plan under the adult aged ≥ 65 years programme. Women were more likely than men to use only antihypertensive medication, whereas men were more likely to be using both BP- and cholesterol-lowering medications. In the two groups, included participants were mostly using only one medication at baseline. Finally, women were slightly more likely than men to experience no change in their pharmacotherapy over the 12-month period preceding FFQ completion, even though, in the two groups, medication remained stable for more than 80 % of the participants.

**Table 1.** Gender-specific characteristics of the 472 participants included in the study^*^ (Numbers and percentages; mean values and standard deviations)

	Women (*n* 268)		Men (*n* 204)	
Characteristics	Mean	sd	Mean	sd
Age (years)	62·7	5·9	62·1	6·3
	*n*	%	*n*	%
Annual household income, *n* (%)				
< $50 000	172	64·2	109	53·4
$50 000 < $100 000	75	28·0	69	33·8
≥ $100 000	21	7·8	26	12·8
Smoking status, *n* (%)				
Never	122	45·5	70	34·3
Past	101	37·7	98	48·0
Current	45	16·8	36	17·7
	Mean	sd	Mean	sd
BMI (kg/m²)	29·1	5·9	28·8	4·4
	*n*	%	*n*	%
Physical activity level, *n* (%)				
Low	52	19·4	31	15·2
Moderate	103	38·4	64	31·4
High	113	42·2	109	53·4
Energy intake (kcal/d)	1760	589	2017	665
	Mean	sd	Mean	sd
Alcohol consumption (grams/d)	6·4	9·5	15·4	25·6
	*n*	%	*n*	%
Québec Public Prescription Drug Insurance Plan programme, *n* (%)				
Adult aged < 65 years	36	13·4	38	18·6
Adult aged ≥ 65 years	147	54·9	128	62·8
Adult receiving financial assistance	85	31·7	38	18·6
Pharmacotherapy type, *n* (%)				
Antihypertensive medication only	123	45·9	70	34·3
Cholesterol-lowering medication only	77	28·7	55	27·0
Both	68	25·4	79	38·7
Number of cardiopreventive medications claimed at baseline, *n* (%)				
1	180	67·2	114	55·9
2	61	22·8	65	31·9
≥ 3	27	10·1	25	12·3
Stable cardiopreventive pharmacotherapy, *n* (%)	237	88·4	169	82·8

* Continuous variables are presented as mean (sd). Categorical variables are presented as count (percent).

**Figure 1.**
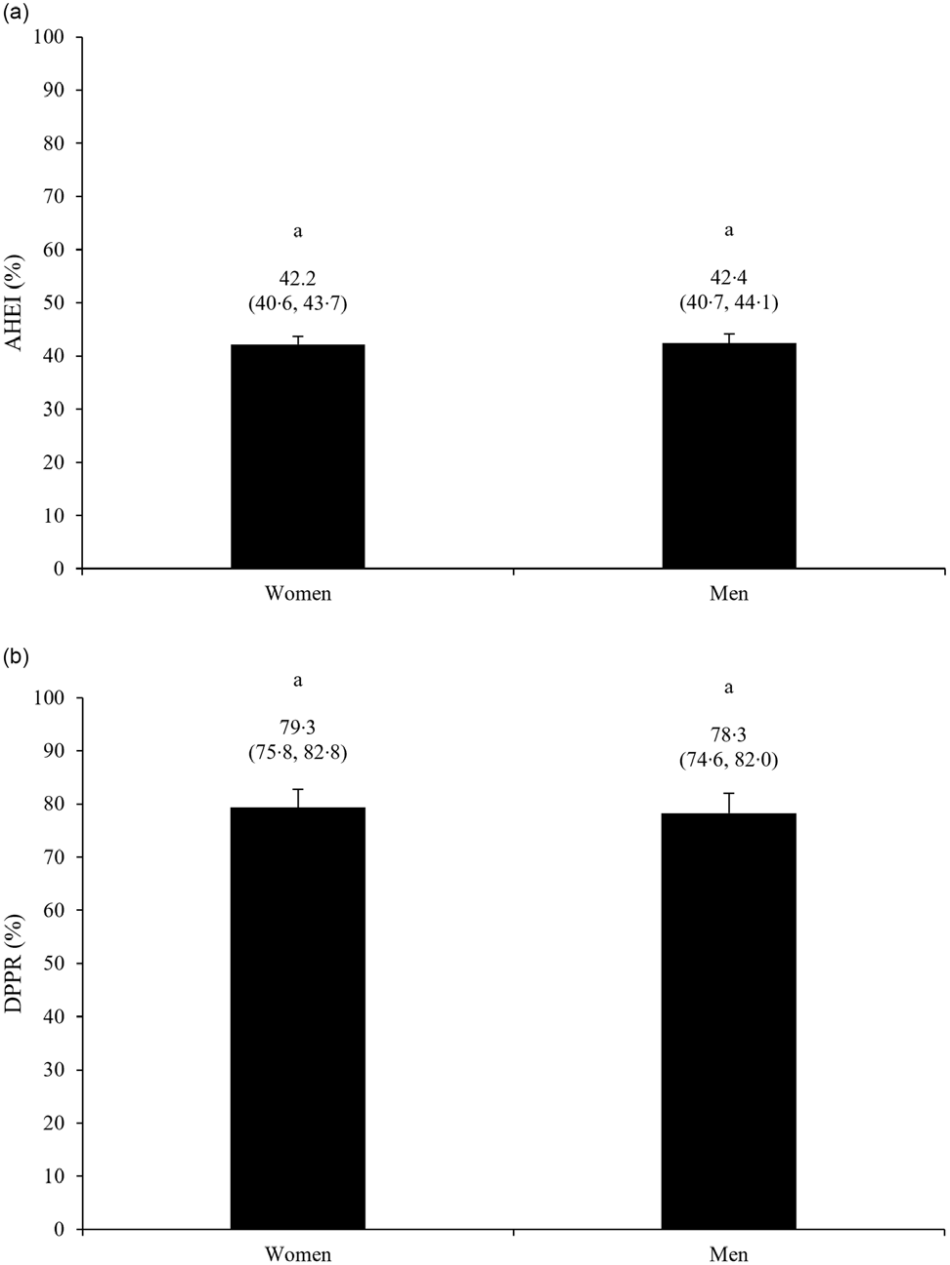
Differences in (a) Alternate Healthy Eating Index (AHEI) and (b) daily pharmacotherapy possession rate (DPPR) between women and men. Data are presented as mean (95 % CI). Models were adjusted for age (years), annual household income (< $50 000; $50 000 < $100 000; ≥ $100 000), BMI (kg/m^2^), smoking status (never, past and current), alcohol consumption (grams/d), energy intake (kcal/d), physical activity level (low, moderate and high) and pharmacotherapy type (antihypertensive medication and cholesterol-lowering medication). Model for difference in AHEI was adjusted for DPPR and vice versa. Columns with different letters are statistically different (*P* < 0·05). *P*_AHEI_ = 0·78; *P*_DPPR_ = 0·63.

We first compared both AHEI and DPPR between women and men. After adjustment for confounding variables, no evidence of a difference in AHEI and DPPR was found between women and men, respectively (Figure 1). Main analyses focused on potential differences in the association between adherence to healthy diet principles and adherence to cardiopreventive medication (Table 2), and we observed that this association significantly differed between women and men (*P*_DPPR×gender_ = 0·01). Among men, the AHEI was inversely associated with DPPR (ΔAHEI for each 10 % increment in DPPR = –0·65 %; 95 % CI −1·28 %, −0·03 %; *P* = 0·04), while the association tended to be positive among women (ΔAHEI for each 10 % increment in DPPR = 0·44 %; 95 % CI −0·11 %, 1·00 %; *P* = 0·12). The same results were obtained when adherence to cardiopreventive medication was assessed using the two alternative DPPR calculation methods (online Supplementary Table S1). No evidence of difference in AHEI was found when participants were grouped according to the three thresholds of medication adherence (online Supplementary Figure S3).

**Table 2.** Gender-specific associations between adherence to healthy diet principles, assessed using the AHEI, and adherence to cardiopreventive medication, assessed using the DPPR^*^ (Beta coefficients and 95 % confidence intervals)

AHEI components	Women (*n* 268)			Men (*n* 204)			*P* for interaction
	*β*	95 % CI	*P*	*β*	95 % CI	*P*	
Total score (%)	0·44	–0·11, 1·00	0·12	–0·65	–1·28, −0·03	0·04	0·01
Sub-scores, /10 points							
Vegetables	–0·01	–0·13, 0·12	0·93	–0·03	–0·17, 0·10	0·62	0·76
Fruits^†^	0·02	–0·11, 0·15	0·92	–0·14	–0·29, 0·01	0·01	0·03
Whole grains^‡^	0·02	–0·07, 0·11	0·93	–0·13	–0·23, −0·02	0·05	0·15
Nuts and legumes^‡^	–0·01	–0·16, 0·15	0·47	–0·07	–0·25, 0·11	0·55	0·98
Fatty fish^†^	0·21	0·02, 0·41	0·18	–0·10	–0·33, 0·13	0·81	0·29
PUFA	–0·00	–0·09, 0·09	0·97	–0·02	–0·12, 0·09	0·72	0·80
Sugar-sweetened beverages^†^,^§^	0·14	–0·08, 0·35	0·11	0·03	–0·21, 0·28	0·90	0·34
Red or processed meat^§^	–0·01	–0·14, 0·12	0·83	0·15	0·06, 0·30	0·04	0·09
*Trans*-fat^§^	0·04	–0·03, 0·11	0·28	–0·03	–0·11, 0·05	0·47	0·21
Na^†^,^§^	–0·04	–0·13, 0·05	0·43	–0·08	–0·19, 0·02	0·10	0·48

AHEI, Alternate Healthy Eating Index; DPPR, daily pharmacotherapy possession rate.

* Data are presented as *β* coefficients (95 % CI) reflecting the difference in AHEI total score (%) or AHEI sub-scores (/10 points) associated with a 10 % increment in DPPR. Models were adjusted for age (years), annual household income (< $50 000; $50 000 < $100 000; ≥ $100 000), BMI (kg/m^2^), smoking status (never, past and current), alcohol consumption (grams/d), energy intake (kcal/d), physical activity level (low, moderate and high) and pharmacotherapy type (antihypertensive medication, cholesterol-lowering medication and both) and included the interaction term between DPPR and gender. Models for AHEI sub-scores were mutually adjusted for each sub-score.

† Required rank transformation. Untransformed *β* coefficients and CI are presented, along with *P*-values calculated from the transformed model.

‡ Required log transformation. Untransformed *β* coefficients and CI are presented, along with *P*-values calculated from the transformed model.

§ Higher sub-scores reflect lower intakes.

AHEI sub-score analyses allowed us to identify that the difference in the direction of the association between AHEI and DPPR between women and men was mostly due to whole fruit intake. Indeed, among men only, AHEI sub-score for whole fruit intake was inversely associated with the DPPR (Table 2). Similarly, in men, AHEI sub-score for whole grain intake was negatively associated with medication adherence, but the interaction test assessing whether this association differed between women and men did not reach statistical significance (*P*_Gender×DPPR_ = 0·18). Finally, among men, AHEI sub-score for red or processed meat consumption was positively associated with the DPPR, meaning that the consumption was negatively associated with medication adherence. The *P*-value of the interaction test assessing whether this association differed relative to women was 0·09.

Among women, stratified analyses allowed the identification of subgroups among which DPPR and AHEI were significantly correlated (Table 3). Among women with a BMI ≥ 30 kg/m^2^, DPPR and AHEI were positively correlated (ΔAHEI for each 10 % increment in DPPR = 1·05 %; 95 % CI 0·12 %, 1·98 %, *P* = 0·03), but no evidence of an association was observed among those with a lower BMI (*P*_interaction_ = 0·06). Likewise, among those who used ≥ 3 medications at some point during the 12-month study period, the AHEI was positively correlated with the DPPR (ΔAHEI for each 10 % increment in DPPR = 2·35 %; 95 % CI 0·03, 4·66, *P* = 0·05), but not among those who used one or two medications (*P*_interaction_ = 0·08). Finally, statistical trends suggested that, among women who were active smokers, adherence to healthy diet principles was negatively associated with adherence to medication (ΔAHEI for each 10 % increment in DPPR = -1·52 %; 95 % CI −3·33, 0·30, *P*_interaction_ = 0·11). Among men, stratified analyses did not reveal subgroups among which the association between the DPPR and the AHEI differed, as all estimates were suggestive of a negative association and no evidence of statistical interactions was documented (Table 4). Still, the negative association between AHEI and DPPR appeared stronger (i.e. absolute ΔAHEI for each 10 % increment in DPPR > 1·00 %) among those who never smoked and those using cholesterol-lowering medication only.

**Table 3.** Association between adherence to healthy diet principles, assessed using the AHEI, and adherence to cardiopreventive medication, assessed using the DPPR, among women (*n* 268), after stratification by key characteristics^*^ (Beta coefficients and 95 % confidence intervals)

Stratification	*β*	95 % CI	*P*	*P* for interaction
Education level				
High school or less (*n* 84)	0·46	–0·65, 1·55	0·42	0·78
College or university (*n* 184)	0·28	–0·33, 0·89	0·37	
Annual household income				
< $50 000 (*n* 172)	0·46	–0·19, 1·11	0·16	0·44
≥ $50 000 (*n* 96)	0·02	–0·92, 0·95	0·97	
BMI				
< 30 kg/m^2^ (*n* 170)	–0·02	–0·67, 0·62	0·94	0·06
≥ 30 kg/m^2^ (*n* 98)	1·05	0·12, 1·98	0·03	
Smoking status				
Never (*n* 122)	0·40	–0·28, 1·08	0·25	0·11
Past (*n* 101)	0·68	–0·33, 1·68	0·19	
Current (*n* 45)	–1·52	–3·33, 0·30	0·10	
Pharmacotherapy type				
Antihypertensive medication only (*n* 123)	0·34	–0·40, 1·09	0·37	0·83
Cholesterol-lowering medication only (*n* 77)	0·12	–0·80, 1·04	0·79	
Both (*n* 68)	0·64	–0·81, 2·09	0·38	
Maximum number of cardiopreventive medications dispensed during the study.				
≤ 2 (*n* 231)	0·24	–0·31, 0·78	0·40	0·08
≥ 3 (*n* 37)	2·35	0·03, 4·66	0·05	
Stability of pharmacotherapy				
Stable (*n* 237)	0·39	–0·16, 0·94	0·17	0·28
Not stable (*n* 31)	–1·07	–3·64, 1·50	0·41	

AHEI, Alternate Healthy Eating Index; DPPR, daily pharmacotherapy possession rate.

* Data are presented as *β* coefficients (95 % CI) reflecting the difference in the AHEI associated with a 10 % increment in DPPR. Models were adjusted for age (years), annual household income (< $50 000; $50 000 < $100 000; ≥ $100 000), BMI (kg/m^2^), smoking status (never, past and current), alcohol consumption (grams/d), energy intake (kcal/d), physical activity level (low, moderate and high) and pharmacotherapy type (antihypertensive medication and cholesterol-lowering medication). Models also included the interaction term between DPPR and the stratification variable.

**Table 4.** Association between adherence to healthy diet principles, assessed using the AHEI, and adherence to cardiopreventive medication, assessed using the DPPR, among men (*n* 204), after stratification by key characteristics^*^ (Beta coefficients and 95 % confidence intervals)

Stratification	*β*	95 % CI	*P*	*P* for interaction
Education level				
High school or less (*n* 52)	–0·50	–2·03, 1·04	0·52	0·99
College or university (*n* 152)	–0·50	–1·21, 0·21	0·16	
Annual household income				
< $50 000 (*n* 109)	–0·20	–1·17, 0·77	0·68	0·34
≥ $50 000 (*n* 95)	–0·83	–1·72, 0·05	0·06	
BMI				
< 30 kg/m^2^ (*n* 138)	–0·63	–1·40, 0·14	0·11	0·63
≥ 30 kg/m^2^ (*n* 66)	–0·27	–1·52, 0·98	0·67	
Smoking status				
Never (*n* 70)	–1·00	–1·99, −0·01	0·05	0·47
Past (*n* 98)	–0·23	–1·25, 0·79	0·65	
Current (*n* 36)	–0·08	–1·69, 1·53	0·92	
Pharmacotherapy type				
Antihypertensive medication only (*n* 70)	–0·07	–1·13, 0·98	0·89	0·24
Cholesterol-lowering medication only (*n* 55)	–1·30	–2·41, −0·19	0·02	
Both (*n* 79)	–0·24	–1·46, 0·97	0·69	
Maximum number of cardiopreventive medication dispensed during the study				
≤ 2 (*n* 176)	–0·61	–1·28, 0·06	0·07	0·55
≥ 3 (*n* 28)	0·12	–2·21, 2·44	0·92	
Stability of pharmacotherapy				
Stable (*n* 169)	–0·63	–1·32, 0·06	0·07	0·83
Not stable (*n* 35)	–0·40	–2·37, 1·57	0·69	

AHEI, Alternate Healthy Eating Index; DPPR, daily pharmacotherapy possession rate.

* Data are presented as *β* coefficients (95 % CI) reflecting the difference in the AHEI associated with a 10 % increment in DPPR. Models were adjusted for age (years), annual household income (< $50 000; $50 000 < $100 000; ≥ $100 000), BMI (kg/m^2^), smoking status (never, past and current), alcohol consumption (grams/d), energy intake (kcal/d), physical activity level (low, moderate and high) and pharmacotherapy type (antihypertensive medication and cholesterol-lowering medication). Models also included the interaction term between DPPR and the stratification variable.

## Discussion

In this cross-sectional study nested within the CARTaGENE Quebec population-based cohort, we observed that the association between adherence to healthy diet principles and adherence to cardiopreventive medication differed between women and men with hypertension and/or dyslipidemia. Indeed, among men, adherence to healthy diet principles was negatively associated with adherence to cardiopreventive medication, whereas among women, these two adherence behaviours tended to be positively correlated. Specifically, among women with obesity or using three or more cardiopreventive medications, this positive association was stronger and statistically significant. Overall, our results suggest that association between adherence to healthy diet principles and to cardiopreventive medication differs between women and men with hypertension and/or dyslipidemia, with men potentially facing greater challenges in achieving optimal complementarity between these two behaviours. Such data are important to inform the development of gender-specific targeted interventions promoting long-term co-adherence to healthy diet principles and medication for optimal primary prevention of CVD.

In men using cardiopreventive medication, adherence to healthy diet principles and to medication were inversely associated, meaning that men who exhibited greater adherence to medication concomitantly adhered less to healthy diet principles and vice versa. We acknowledge that, at the sample level, the clinical significance of this observation can be questioned given that the effect size is relatively small, with each 10 % increment in DPPR associated with a −0·65 % difference in AHEI. Despite the small effect size, the demonstration of a negative association between these two adherence behaviours among men is of clinical significance. Indeed, this finding underscores the need to address dietary and pharmacological adherence jointly rather than independently in counselling. Expanding on our previous findings within the same cohort, which showed that among men with metabolic syndrome, those using a statin had a AHEI 1·8 % lower compared with non-users of this cholesterol-lowering medication^(14)^, we cannot exclude that the negative association between adherence to healthy diet principles and to medication may further exacerbate the difference in diet quality associated with medication use. For broader contextualisation of the clinical significance of our results, it is worth noting that, among adults from the province of Québec, between 2004 and 2015, adherence to healthy diet principles, as assessed by the AHEI, increased by only 1·6 %^(17)^. As such, assuming causality in our observations, one could suggest that the increase in the prevalence of cardiopreventive medication use over the past decade in Québec and Canada could have hinder public health efforts to improve diet quality among adult men with hypertension and/or dyslipidemia^(47–50)^. Besides, although interaction tests did not reveal evidence of statistically different association between adherence to healthy diet principles and to medication among specific subgroups of men, these analyses still warrant discussion. Indeed, the negative association between the two adherence behaviours of interest appeared stronger – as showed by greater *β* coefficients – among men with a higher annual household income, those who never smoked, those who only used a cholesterol-lowering medication, those who used less than two medications and those who maintained a stable pharmacotherapy over the 12-month study period. These characteristics may all be associated with an overall lower perceived and/or objectified risk of CVD^(1)^. As such, these observations suggest potential social biases in how CVD risk is perceived and how the effectiveness of medication and diet is comparatively considered not only among men but also among healthcare providers interacting with them. Medication is generally perceived by both healthcare providers and men as more effective and easier to implement than dietary changes^(51–55)^. Such biases are likely to reduce opportunities and motivation to engage in dietary management when initiating cardiopreventive medication^(56)^. Hence, the current study adds to the body of evidence highlighting the challenges of adopting a truly complementary approach between medication use and healthy dieting for optimal CVD prevention among men with prevalent hypertension and/or dyslipidemia. Moreover, identifying subgroups of men with distinct characteristics where the negative association between medication and dietary adherence is stronger holds further clinical significance. This can aid in screening individuals who are likely to benefit more from joint counselling on both behaviours.

In women using cardiopreventive medication, adherence to healthy dietary principles and medication tended to be positively associated in the overall sample. However, stronger, statistically significant positive associations were observed among women with obesity and those concurrently using three or more medications. Again, interaction tests did not reach statistical significance, but these observations also warrant mention, especially since they reflect stronger complementary adherence behaviours among women with a higher risk of CVD. When analysed alongside the results observed among men, a clinically significant pattern emerges: among men, a lower CVD risk profile appears associated with a stronger negative association between adherence to healthy diet principles and to cardiopreventive medication. Conversely, among women, a higher risk profile is associated with greater alignment in adherence to healthy diet and cardiopreventive medication. As such, beyond effect size, we believe the primary clinical takeaway of our findings is that women and men differ in their co-adherence behaviours related to healthy dietary principles and cardiopreventive medications. This suggests that counselling on diet and medication should be gender-specific to optimise the complementarity between dietary and pharmacological prevention of CVD. In perspective, these observations also raise legitimate questions on how medication is differentially perceived and used relative to diet by both women and men in CVD prevention schemes. Our data suggest that it could be positioned as a substitute or compensatory modality relative to diet among men, while, among women, it would be perceived as a complementary preventive modality.

As previously mentioned, it has been repeatedly reported that women exhibit slightly greater adherence to healthy diet principles, but lower adherence to cardiopreventive medication relative to men^(17,18,20–23)^. These differences could be attributable to a greater preference towards lifestyle modification relative to medication interventions among women with hypertension and/or dyslipidemia^(24,57)^. In our study, we did not observe evidence of differences in adherence to healthy diet principles, assessed using the AHEI, and adherence to cardiopreventive medication, evaluated with the DPPR, between women and men. For healthy diet principle adherence, we cannot exclude insufficient statistical power, especially since previous analyses conducted in the Canadian Community Healthy Survey, comprising between 20 000 and 35 000 individuals, reported a AHEI 2 % to 3 % higher in women compared with men^(17)^. Still, with a mean AHEI of 42 % in both women and men in our sample, overall adherence to healthy diet principles aligned with estimates previously reported in cohorts from Québec^(58)^, Canada^(17)^, and elsewhere in North America^(19)^. For medication adherence, we cannot exclude that coverage under the Québec Public Prescription Drug Insurance Plan removed gender-specific barriers to medication (e.g. economic cost) leading to similar adherence between women and men, as previously reported in other studies conducted in Québec^(59)^. More broadly, while the AHEI and the DPPR reflect distinct adherence behaviours and cannot be formally compared, it is worth noting that, in our sample, adherence to healthy diet principles fell within the highly suboptimal range, whereas with mean DPPR just below 80 % in both women and men, our sample could be considered as borderline adherent to their cardiopreventive pharmacotherapy^(46)^. As such, our study reinforces the importance of removing systemic and individual barriers to preventive modalities, especially adherence to healthy diet principles.

We could not assess how the association between adherence to healthy diet principles and to cardiopreventive medication translated into plasma cholesterol or BP control, given the lag between physical assessments (2009–2010) and diet and medication adherence measurements (2011–2012) in the CARTaGENE cohort. Still, it has been reported that, in both Canada and Québec, the rate of adequate control of dyslipidemia remains largely insufficient, while hypertension control has even declined since 2010, in spite of important increases in the prevalence of use of cholesterol- and BP-lowering medications^(60,61)^. Even though cardiopreventive medications are highly effective in controlling CVD risk factors, it cannot be excluded that suboptimal control of CVD risk factors at the population level can be attributed to inadequate complementarity between pharmacological and lifestyle preventive approaches, as observed in the current and in our previous studies^(12–14)^. Besides, long-term adherence to cholesterol- and BP-lowering medications in primary prevention of CVD is known to be highly suboptimal^(47)^. For instance, in the province of Québec, only 53 % of individuals covered by the Québec Public Prescription Drug Insurance Plan and using a statin are considered adherent after 5 years of treatment initiation, and about 17 % are no longer persistent^(47)^. This issue highlights the crucial importance of reinforcing adherence to lifestyle and diet modification as complements to cardiopreventive medication. In that regard, the interpretation of our results is limited by the lack of information on the history of diet counselling among the study participants. This information was not collected in CARTaGENE health questionnaire, and it is also not compiled in Québec Health Administrative databases. In 2012, a pan-Canadian survey on CVD risk management in primary care revealed concerning information about access to diet counselling among at-risk individuals. Indeed, only 21 % of respondents (i.e. users of primary care) reported having received dietary counselling, albeit being at risk of CVD^(62)^. Conversely, rates of cholesterol- or BP-lowering medication use were much higher, ranging from 75 % to 92 %. Over the past decade, it remains unclear whether access to diet counselling has improved in the province of Québec. Indeed, an audit of clinical notes in an outpatient cardiology clinic in Montréal (Québec, Canada) revealed that less than 16 % of patients followed at this clinic received diet counselling in 2019^(63)^. As such, to improve complementarity in dietary and pharmacological prevention of CVD, facilitating access to lifestyle health professionals needs to be both monitored and reinforced. Additionally, in line with our main results, we cannot exclude the possibility that differences may exist in the association between adherence to healthy dietary principles and medication adherence between today and when the data were collected. However, our findings highlight the need to consider this interrelationship to optimise cardiopreventive strategies, independent of the situation being better or worse relative to early 2010s.

The methodological limitations of our work include, first, the use of the FFQ to assess diet quality, which inevitably involves measurement errors, despite the validation of the CDHQII^(38)^. For medication adherence, we used an objective and validated prescription claim-based method; however, these data reflect medication possession rather than actual consumption and must be interpreted accordingly^(46)^. Additionally, claim data do not distinguish between non-adherence and healthcare professional-recommended medication cessation. Furthermore, we cannot exclude the possibility that dosages may act as undocumented effect modifiers in our results through dose-dependent risks of adverse effects. Nonetheless, our results can still be interpreted as valid independent of dosage variations. We chose to position this work within the early stages of primary prevention of CVD, excluding individuals with prevalent CVD, diabetes and/or cancer. While this approach reduced potential confounding by co-morbidities, it also narrowed the target population to individuals with prevalent hypertension and/or dyslipidemia. We also cannot exclude that some of our analyses were underpowered as several statistical trends were observed. In that regard, we had limited, if any, control over sample size, especially since we believe our choice of inclusion and exclusion criteria are well justified to position this work within primary prevention of CVD. Technically, *a priori* power calculation could not be conducted because no prior study has assessed the association between the AHEI and the DPPR, leaving no data to inform potential strength of the association. Conceptually, we believe it is important to highlight that, in the present context, achieving higher power would have allowed us to detect statistical significance in very weak associations, which may not have much clinical or public health relevance. The main strength of our analysis relies on the co-assessment of adherence to healthy diet principles and to medication over a 12-month period. Indeed, previous studies conducted on the same topic assessed adherence to both healthy diet principles and medication using questionnaires with unclear time frames^(15,16)^.

### Conclusions

In this cohort of adults from Québec, we observed that the association between adherence to healthy diet principles and cardiopreventive medication differs between women and men with hypertension and/or dyslipidemia. Among men, these two adherence behaviours were negatively correlated, while among women, these two adherence behaviours tended to be positively correlated. These data suggest that optimal complementarity between adherence to healthy diet principles and to cardiopreventive medication may be more challenging to achieve among men than women. While the long-term implications of these results relative to CVD prevention warrant future studies, they may readily inform the development of gender-specific targeted interventions promoting long-term adherence to healthy diet principles and medication for optimal primary prevention of CVD.

## Supporting information

Leblay et al. supplementary material Leblay et al. supplementary material

## Supplementary material

For supplementary material/s referred to in this article, please visit https://doi.org/10.1017/S0007114525000030.
