# Supplementary material for: Gender differences in the association between adherence to healthy diet principles and adherence to cardiopreventive medication among adults from Québec (Canada)
Source: Br J Nutr. 2025 Jan 16;133(3):422–32. doi: 10.1017/S0007114525000030 (PMC11946037; doi:10.1017/S0007114525000030)
Supplement: Leblay et al. supplementary material [file S0007114525000030sup001.docx]

**Supplemental material**

**Gender differences in the association between adherence to healthy diet principles and adherence to cardiopreventive medication among adults from Québec (Canada).**

Lise Leblay, Jacob Lessard-Lord, Jean-Sébastien Paquette, Line Guénette, Jean-Philippe Drouin-Chartier

**Corresponding author**:

Jean-Philippe Drouin-Chartier

[jean-philippe.drouin-chartier@pha.ulaval.ca](mailto:jean-philippe.drouin-chartier@pha.ulaval.ca)

**Supplemental Table S1: Differential association between AHEI and medication adherence, according to the participants’ gender and alternative medication adherence calculation approaches.**

| **AHEI components** | **Women (n=268)** | | **Men (n=204)** | | ***P* value for interaction** |
| --- | --- | --- | --- | --- | --- |
|  | **β (95% CI)** | ***P* value** | **β (95% CI)** | ***P* value** |  |
| DPPR based on full pharmacotherapy availability | 0.43  (-0.12, 0.98) | 0.12 | -0.63  (-1.27, 0.01) | 0.05 | 0.01 |
| DPPR based on partial pharmacotherapy availability | 0.43  (-0.08, 0.95) | 0.10 | -0.61  (-1.17, -0.5) | 0.03 | 0.01 |

*^1^* Data are presented as β coefficients (95% confidence interval) reflecting the difference in AHEI (%) associated with a 10% increment in DPPR. Models were adjusted for age (years), annual household income (<$50,000; $50,000<$100,000; ≥$100,000), BMI (kg/m^2^), smoking status (never, past, current), alcohol consumption (grams/day), energy intake (kcal/day), physical activity level (low, moderate, high), and pharmacotherapy type (antihypertensive medication, cholesterol-lowering medication, both), and included the interaction term between DPPR and gender.


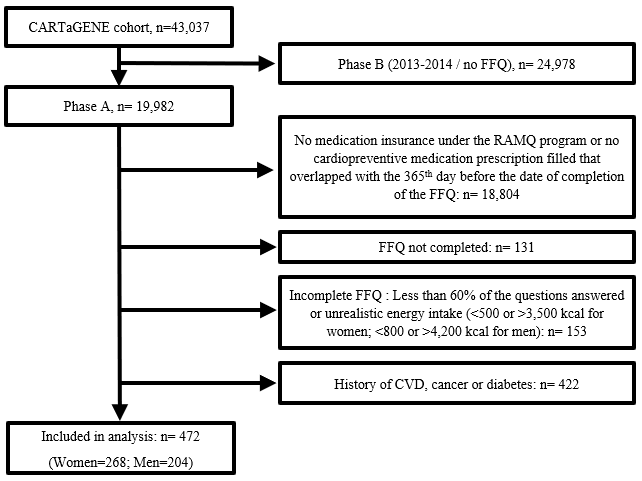
**Supplemental Figure S1: Flow-chart of participants’ selection.**

**
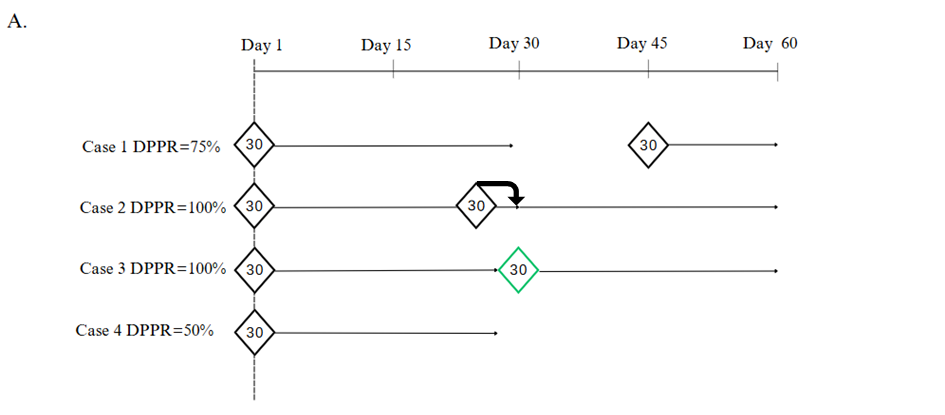
**

**
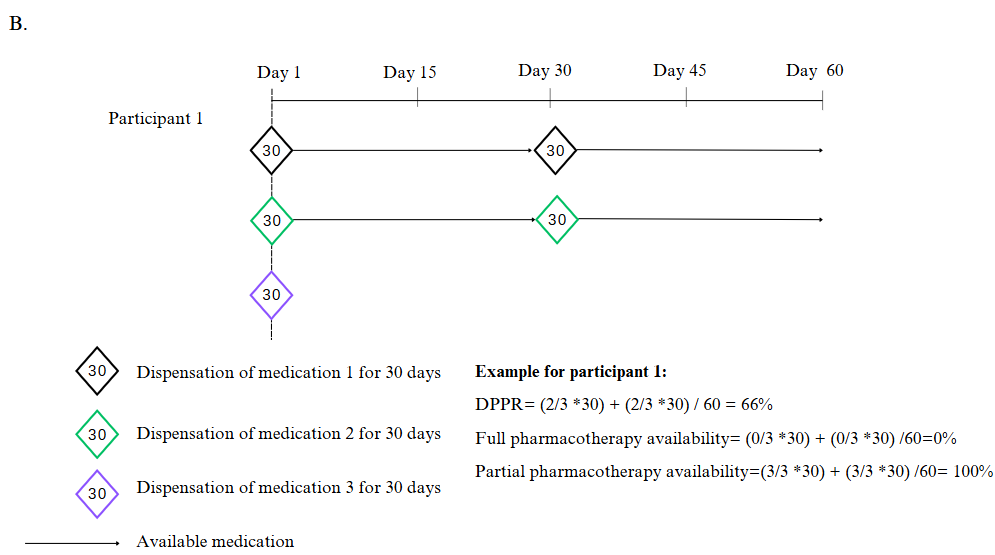
**

**Supplemental Figure S2:** **Illustration of examples of DPPR calculation.**


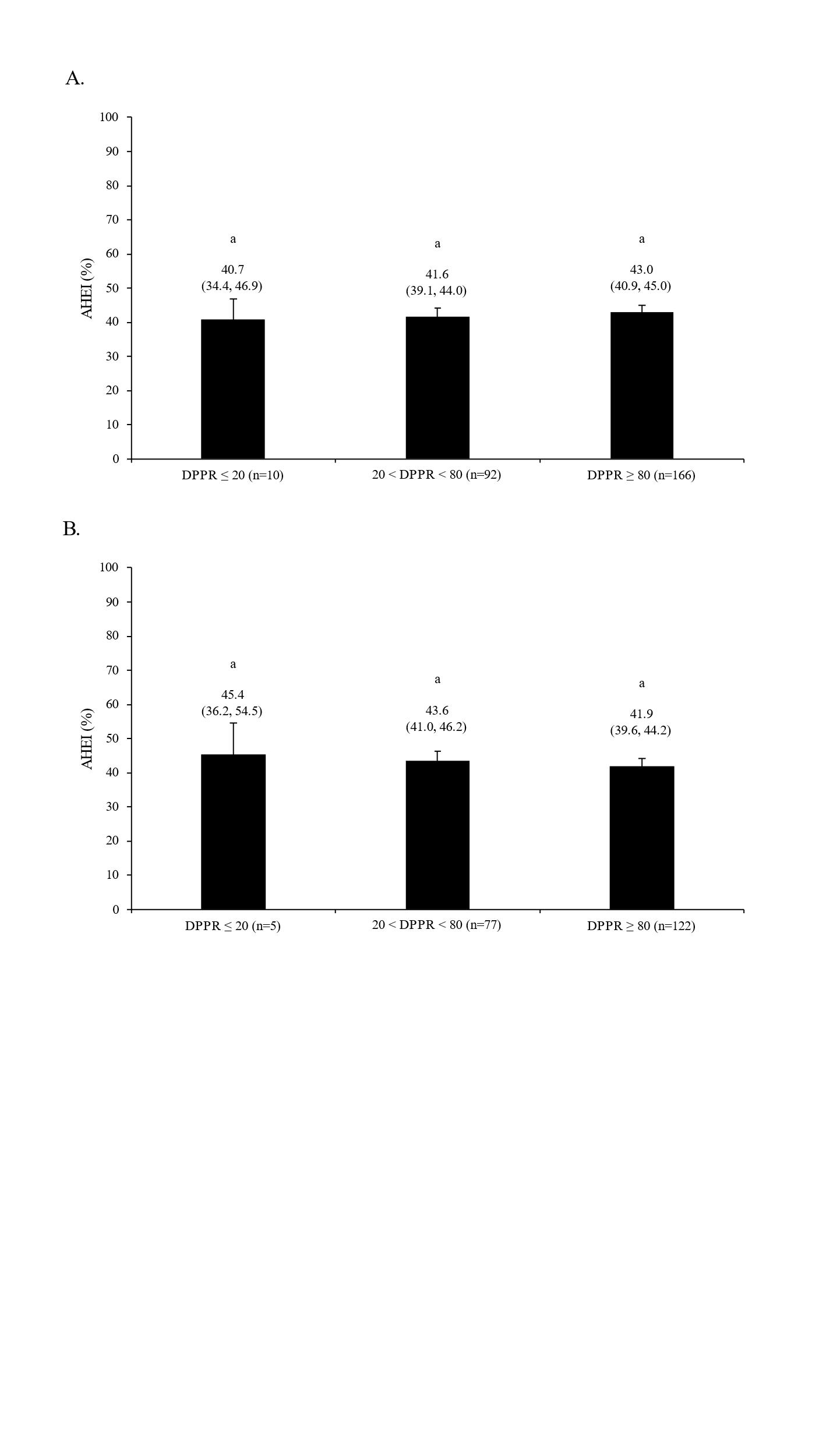


**Supplemental Figure S3: Alternate healthy eating index (AHEI) according to 3 thresholds of adherence to cardiopreventive medication among women (A) and men (B).** Data are presented as mean (95% confidence interval). Models were adjusted for age (years), annual household income (<$50,000; $50,000<$100,000; ≥$100,000), BMI (kg/m^2^), smoking status (never, past, current), alcohol consumption (grams/day), energy intake (kcal/day), physical activity level (low, moderate, high), and pharmacotherapy type (antihypertensive medication, cholesterol-lowering medication). Columns with different letters are statistically different (*P*<0.05).
